# 

**Published:** 1909-01

**Authors:** 


The fifth semi-annual meeting of the Chattahoochee Valley Medical and Surgical Association will be held at Opelika, Ala., January 12th and 13th. Quite an interesting program has been arranged.

